# Correction to: Fostamatinib in chronic immune thrombocytopenia: a profile of its use in the USA

**DOI:** 10.1007/s40267-018-0575-2

**Published:** 2018-11-16

**Authors:** Kate McKeage, Katherine A. Lyseng-Williamson

**Affiliations:** Springer, Private Bag 65901, Mairangi Bay, Auckland, 0754 New Zealand

## Correction to: Drugs & Therapy Perspectives (2018) 34(10):451–456 10.1007/s40267-018-0551-x

The article Fostamatinib in chronic immune thrombocytopenia: a profile of its use in the USA, was originally published Online First without open access. After publication in volume 34, issue 10, pages 451–456, Rigel Pharmaceuticals, Inc. requested that the article be Open Choice to make the article an open access publication. Post-publication open access was funded by Rigel Pharmaceuticals, Inc. The article is forthwith distributed under the terms of the Creative Commons Attribution-NonCommercial 4.0 International License (http://creativecommons.org/licenses/by-nc/4.0/), which permits any noncommercial use, duplication, adaptation, distribution and reproduction in any medium or format, as long as you give appropriate credit to the original author(s) and the source, provide a link to the Creative Commons license and indicate if changes were made.

The original article has been corrected.

